# Investigation of Biogas Dry Reforming over Ru/CeO_2_ Catalysts and Pd/YSZ Membrane Reactor

**DOI:** 10.3390/membranes16010034

**Published:** 2026-01-05

**Authors:** Omid Jazani, Simona Liguori

**Affiliations:** Chemical and Biomolecular Engineering Department, Clarkson University, Potsdam, NY 13699, USA; jazanio@clarkson.edu

**Keywords:** Pd-based membrane reactor, dry reforming reaction, Ru/CeO_2_ catalyst, hydrogen production

## Abstract

The biogas dry reforming reaction offers a promising route for syngas production while simultaneously mitigating greenhouse gas emissions. Membrane reactors have proven to be an excellent option for hydrogen production and separation in a single unit, where conversion and yield can be enhanced over conventional processes. In this study, a Pd/YSZ membrane integrated with a Ru/CeO_2_ catalyst was evaluated for biogas reaction under varying operating conditions. The selective removal of hydrogen through the palladium membrane improved reactant conversion and suppressed side reactions such as methanation and the reverse water–gas shift. Experiments were performed at temperatures ranging from 500 to 600 °C, pressures of 1–6 bar, and a gas hourly space velocity (GHSV) of 800 h^−1^. Maximum conversions of CH_4_ (43%) and CO_2_ (46.7%) were achieved at 600 °C and 2 bar, while the maximum hydrogen recovery of 78% was reached at 6 bar. The membrane reactor outperformed a conventional reactor, offering up to 10% higher CH_4_ conversion and improved hydrogen production and yield. Also, a comparative analysis between Ru/CeO_2_ and Ni/Al_2_O_3_ catalysts revealed that while the Ni-based catalyst provided higher CH_4_ conversion, it also promoted methane decomposition reaction and coke formation. In contrast, the Ru/CeO_2_ catalyst exhibited excellent resistance to coke formation, attributable to ceria’s redox properties and oxygen storage capacity. The combined system of Ru/CeO_2_ catalyst and Pd/YSZ membrane offers an effective and sustainable approach for hydrogen-rich syngas production from biogas, with improved performance and long-term stability.

## 1. Introduction

The continuous rise in global energy demand, alongside the escalating challenges of climate change, highlights the necessity for sustainable and low-carbon energy carriers [1,2,3,4]. Hydrogen (H_2_) has gained considerable attention as a clean energy vector capable of enabling decarbonization across different sectors, including transportation, power generation, and chemical manufacturing [5,6,7]. At present, hydrogen production is predominantly reliant on steam methane reforming (SMR), a process that is both energy-intensive and a major source of CO_2_ emissions. Consequently, the development of renewable-based hydrogen production pathways is critical for achieving climate mitigation targets and reducing dependency on fossil resources. Biogas, composed primarily of methane (CH_4_) and carbon dioxide (CO_2_) generated via the anaerobic digestion of organic substrates such as agricultural residues, municipal solid waste, and sewage sludge, represents a promising renewable feedstock [8,9,10]. Substantial quantities of biogas are already produced at wastewater treatment plants, landfills, and agricultural facilities. However, it is applied in low-efficiency processes such as direct combustion for heat. Reforming biogas to hydrogen offers a higher-value utilization pathway that aligns with circular economy principles by valorizing waste streams, lowering greenhouse gas emissions, and contributing to the production of carbon-neutral fuels. Among the available routes, biogas dry reforming (BDR [MDR and BDR are used interchangeably in this paper when referring to the process and reaction]) represents a promising thermochemical process that simultaneously converts the two most potent greenhouse gases, CO_2_ and CH_4_, into synthesis gas (a mixture of H_2_ and CO) via the so-called methane dry reforming (MDR^1^) reaction [11,12,13,14]. The MDR reaction is highly endothermic, proceeding at elevated temperatures (700–1000 °C) and pressures (15–30 bar), as shown in Equation (1) [15]:

CH_4_ + CO_2_↔2CO + 2H_2_    ΔH°_298_ = 247 kJ/mol (1)

Despite its environmental and industrial advantages, BDR faces several critical challenges. The reaction is thermodynamically limited, leading to partial feed conversion. Additionally, the presence of both CO_2_ and CH_4_ under harsh reaction conditions promotes multiple side reactions (Equations (2)–(8)), including the reverse water–gas shift, methanation, the Boudouard reaction, and methane decomposition, which reduce syngas yield and accelerate carbon formation [13,16,17,18]. Among these, carbon deposition is the most critical issue, as it leads to catalyst deactivation and frequent regeneration cycles. This deactivation inhibits the scalability of BDR despite its promising potential for sustainable syngas production for industrial application [19,20,21]. Overcoming these challenges requires ongoing efforts toward enhanced catalyst design and process intensification strategies to improve both catalyst stability and reactor performance.

Side reactions:

CH_4_ + H_2_O↔CO + 3H_2_    ΔH°_298_ = 206 kJ/mol (methane steam reforming) (2)

CO_2_ + H_2_↔CO + H_2_O    ΔH°_298_ = 41.4 kJ/mol (reverse water–gas shift reaction) (3)

CO_2_ + 4H_2_↔CH_4_ + 2H_2_O    ΔH°_298_ = −165 kJ/mol (methanation reaction) (4)

Carbon formation reactions:

2CO↔CO_2_ + C    ΔH°_298_ = −173 kJ/mol (Boudouard reaction) (5)

CH_4_↔2H_2_ + C    ΔH°_298_ = 75 kJ/mol (methane decomposition) (6)

CO + H_2_↔H_2_O + C    ΔH°_298_ = −131 kJ/mol (carbon formation via hydrogenation reaction) (7)

CO_2_ + 2H_2_↔2H_2_O + C    ΔH°_298_ = −90 kJ/mol (carbon formation via hydrogenation reaction) (8)

So far, both non-noble and noble catalysts have been studied for BDR [22]. Noble metal-based catalysts are particularly attractive due to their exceptional catalytic activity, thermal stability, and superior resistance to carbon deposition. These properties make them highly effective for maintaining long-term catalyst performance under the harsh conditions of dry reforming. However, their high cost and limited availability create significant economic constraints for large-scale applications. In contrast, non-noble metal catalysts, especially Ni-based systems, offer a cost-effective alternative and have demonstrated promising activity and syngas selectivity. Nevertheless, they are generally more susceptible to carbon formation and sintering, which leads to deactivation over time; thus, a more efficient catalyst should be developed. Additionally, the catalyst support plays a critical role that extends beyond providing a structural anchor for the active metal [23,24,25,26]. Among various supports, ceria (CeO_2_) has shown exceptional promise due to its ability to promote uniform metal dispersion and mitigate sintering of active metal particles. Furthermore, its redox properties enable the removal of surface carbon species via lattice oxygen participation. Indeed, CeO_2_ can readily release lattice oxygen through the reduction of Ce^4+^ to Ce^3+^, thereby enhancing the oxidation of intermediate carbon species and improving coke resistance and maintaining catalyst activity during dry reforming reactions [27,28,29,30,31].

While most research has focused on optimizing catalysts within conventional reactors (CRs) to enhance BDR performance and mitigate carbon deposition, integrating the reaction into a hydrogen-selective membrane reactor (MR) represents a promising process intensification strategy. This configuration simultaneously enhances conversion efficiency, hydrogen yield, and product selectivity, while suppressing undesired side reactions such as the reverse water–gas shift (Equation (3)) and methanation (Equation (4)), both of which lower overall system efficiency [14,32,33,34]. By continuously and selectively removing hydrogen from the reaction zone, the MR shifts the reaction equilibrium toward greater syngas formation, achieving higher reactant conversions under milder conditions. Moreover, this in-situ separation suppresses coke formation (Equations (5) and (6)), thereby sustaining catalyst activity and improving the long-term stability and performance of the intensified system [15]. Additionally, the MRs mainly operate at milder conditions than the CRs, which provide some benefits such as reduction in thermal severity, enabling lower material stress, improved membrane stability, and potential integration with decentralized biogas sources. However, the complete conversion could not be achieved at milder operating conditions, and hydrogen recovery in MR mainly depends on pressure [14,32,33,34].

Palladium-based alloy membranes are particularly well-suited for this thermochemical application due to their complete selectivity for hydrogen, thermal stability, and chemical resistance. As a result, Pd-based MRs have been studied for reactions [35,36,37,38]. Sumrunronnasak et al. [39] demonstrated that using a ternary Pd–Ag–Cu MR significantly enhanced both CH_4_ and CO_2_ conversion rates by 30% and 20%, respectively, at 550 °C, when compared to CRs. This was accompanied by an improvement in the H_2_/CO ratio, which increased from 0.6 to 1. Gallucci et al. [32] investigated MDR in a Pd–Ag MR at relatively low temperatures (400–450 °C), reporting 5% CO_2_ conversion and 15% CH_4_ conversion at 450 °C, with minimal impact on conversion when pressure was increased from 1.2 to 2 bar. Galuzka et al. [40] achieved 48% CH_4_ conversion and 63% CO_2_ conversion at 550 °C in a Pd/Al_2_O_3_ MR, outperforming CR (41% CH_4_ conversion, 57% CO_2_ conversion). They also reported an improvement in the H_2_/CO ratio from 0.84 in CR to 0.94 in MR, demonstrating the superior performance of MRs in promoting more efficient reactant consumption and minimizing side reactions. Additional studies by Bosko et al. [41] and Caravella et al. [42] have confirmed that Pd and Pd–Ag MRs offer higher CH_4_ conversion and hydrogen recovery compared to CRs, particularly with the use of Rh/La_2_O_3_ catalysts. Interestingly, Pd–Ag MRs also showed superior hydrogen permeation rates, leading to higher hydrogen recovery and better overall system performance.

The aim of this study is to evaluate the performance of a hydrogen-selective Pd-based MR for BDR reaction under milder operating conditions (500–600 °C, 1–6 bar) using a Ru/CeO_2_ catalyst and an equimolar CH_4_/CO_2_ feed compared with CR. In contrast to conventional biogas compositions (≈60/40 CH_4_/CO_2_), the feed was enriched with CO_2_ captured via Direct Air Capture to establish a closed-loop process that integrates carbon capture with renewable hydrogen generation. A model biogas/CO_2_-enriched without impurities was used to evaluate the intrinsic performance of the catalytic activity and membrane reactor, such as CH_4_ and CO_2_ conversion, hydrogen yield, and recovery. The investigation focuses on assessing how elevated pressure and in-situ hydrogen removal affect CH_4_ and CO_2_ conversions, H_2_ yield and selectivity, suppression of side reactions, and mitigation of carbon deposition. The findings provide new insights into the potential of Pd-based MRs as a viable and sustainable technology for low-carbon hydrogen production from biogas and recycled CO_2_ sources.

## 2. Materials and Methods

The Pd/YSZ composite membrane was manufactured by a thin layer of Pd deposition via electroless plating (ELP) on asymmetric yttria-stabilized zirconia (YSZ) support (4% Y_2_O_3_, 96% ZrO_2_), provided by Praxair Surface Technologies (Indianapolis, IN, USA). The Pd/YSZ membrane was characterized to have an active length of 17.6 cm, an OD of ~1 cm, and a thickness of 13 µm. The total active surface area was measured to be approximately 5.4 × 10^−3^ m^2^.

The Pd/YSZ membrane was packed with a commercial Ru-based catalyst to form MR, which was used for the experimental tests. The MR was heated using an ultra-high temperature heating tape, model STH051-080 (OMEGA Engineering, Norwalk, CT, USA). A thermocouple was inserted into the system via a K-type Omega. Aalborg and Brooks mass flow controllers were used to regulate the feed flow rate. The pressure on the retentate side was controlled with a Swagelok pressure regulator (Swagelok Company, Solon, OH, USA) and measured with a Swagelok EN 837-1 pressure gauge (Swagelok Company, Solon, OH, USA).

The MR was heated with a ramp of 2 °C/min by feeding both sides of the MR with N_2_ gas. After reaching the targeted temperature, pure hydrogen was fed to the system to activate both membrane and catalyst for 24 h. After this step, N_2_ was fed again to check that the membrane was still leak-free. Hydrogen permeation flux was measured using a bubble flow meter during the permeation and reaction tests.

**Figure 1:**
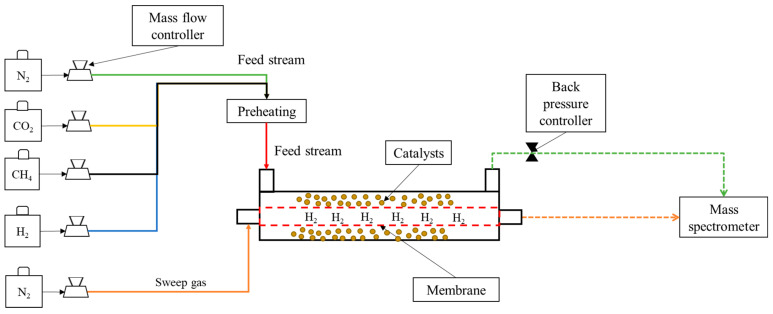
Experimental setup for the BDR reaction performed in Pd/YSZ MR.

A schematic showing the Pd/YSZ MR used to carry out the R experiments is shown in Figure 1.

The membrane was characterized by permeation tests using pure H_2_ at the temperatures of 500, 550, and 600 °C and a pressure range of 1–2.2 bar, and N_2_ leak tests performed up to 6 bar before any reaction tests. Each gas permeating flux through the membrane was calculated using a bubble-flow meter with an average of 10 experimental points and a standard deviation of less than 1%.

The equations used to describe the permeation characteristics of the membrane are reported below:

(9) $$J_{H_{2}}=\;P_{H_{2}}\;\left(p_{H_{2},\mathrm{retentate}}^{n}-p_{H_{2},\mathrm{permeate}}^{n}\right)$$

where J_H_2__ is the hydrogen permeation flux through the membrane, P_H_2__ is hydrogen permeance, $p_{H_{2},\mathrm{retentate}}^{n}$ and $p_{H_{2},\mathrm{permeate}}^{n}$ are hydrogen partial pressures in the retentate and permeate side, respectively, and n is the dependent factor, which shows the correlation between the H_2_ permeation and the driving force; this varies from 0.5 to 1 [43]. Hydrogen permeance depends on temperature and follows the Arrhenius-like equation.

(10) $$P_{H_{2}}=P_{H_{2}}^{0}\;\exp\;(\frac{-\mathrm{Ea}\;}{\mathrm{RT}})$$

$P_{H_{2}}^{0}\;$ is the pre-exponential factor, E_a_ is the apparent activation energy, T is the absolute temperature, and R is the universal gas constant.

Also, hydrogen permeance is defined in Equation (11):

(11) $$P_{H_{2}}=\frac{{\mathrm{Pe}}_{H_{2}}\;}{\delta }$$

where ${\mathrm{Pe}}_{H_{2}}$ is hydrogen permeability, and δ is the membrane thickness.

After the H_2_ permeation tests, the BDR reaction tests were implemented to evaluate the performance of the MR. For the reaction tests, 5.4 g of commercial Ru-based catalyst was packed outside of the membrane, as shown in Figure 1. The equimolar ratio of CH_4_ and CO_2_ was used for the reaction. An Extrel Max-300LG mass spectrometer (MS) was used to evaluate the composition of the permeate and retentate streams for all experimental tests. The MS was tuned and calibrated to ensure accurate molar composition prior to the testing. Each point for the reaction test represents an average of 200 points.

The MR performance has been evaluated by the following equations.

(12) $${\mathrm{CH}}_{4}\;\mathrm{conversion}\;(\%)=\frac{{{\mathrm{CH}}_{4}\;}_{\mathrm{in}}\;-{\;{\mathrm{CH}}_{4}\;}_{\mathrm{out}}}{{{\mathrm{CH}}_{4}\;}_{\mathrm{in}}}\times 100$$

(13) $${\mathrm{CO}}_{2}\;\mathrm{conversion}\;(\%)=\frac{{{\mathrm{CO}}_{2}\;}_{\mathrm{in}}\;-{\;{\mathrm{CO}}_{2}\;}_{\mathrm{out}}}{{{\mathrm{CO}}_{2}\;}_{\mathrm{in}}}\times 100$$

(14) $$H_{2}\;\mathrm{recovery}\;(\%)=\frac{{H_{2}\;}_{\mathrm{permeate}}\;}{{H_{2}\;}_{\mathrm{permeate}\;}+{H_{2}\;}_{\mathrm{retentate}\;}}\times 100$$

Total H_2_ production = H_2 permeate_ + H_2 retentate_ (15)

(16) $$H_{2}\;\mathrm{yield}\;(\%)=\frac{\mathrm{Total}\;H_{2}\;\mathrm{production}}{{2\;{\mathrm{CH}}_{4}\;}_{\mathrm{in}}}$$

where CH_4-in_, CH_4-out_, CO_2-in_, CO_2-out_, H_2-permeate_, and H_2-retentate_ are the inlet and outlet CH_4_, CO_2_, and H_2_ flow rates in the retentate and permeate sides, respectively. The amount of coke was also calculated through a carbon balance (Equation (17)).

Carbon _accumulation(g)_ = Carbon _in(g)_ – Carbon _out(g)_ (17)

Carbon _in(g)_ referred to total grams of carbon entering the MR, and Carbon _out(g)_ referred to the total carbon species (CO_2_, CH_4_, and CO) in the retentate and permeate sides. The carbon balance closure for all experiments was within 95–98%.

Table 1 summarizes the operating conditions used to conduct the BDR reaction in the Pd/YSZ MR.

(18) $$*\mathrm{GHSV}\;(h^{-1})=\frac{\mathrm{Volumetric}\;\mathrm{flow}\;\mathrm{rate}\;\mathrm{of}\;\mathrm{feed}\;\mathrm{gas}\;}{\mathrm{Mass}\;\mathrm{of}\;\mathrm{catalyst}\;\mathrm{bed}\;}$$

where the volumetric flow rate of the feed gas is CH_4_ and CO_2_ total flow rates. Gas-phase carbon flow rates were converted to mass using molar flow rates and molecular weights.

Also, the same tests were performed in the CRs at the same operating conditions to compare the results with the MR performances.

## 3. Results and Discussion

### 3.1. H_2_ Permeation

**Figure 2:**
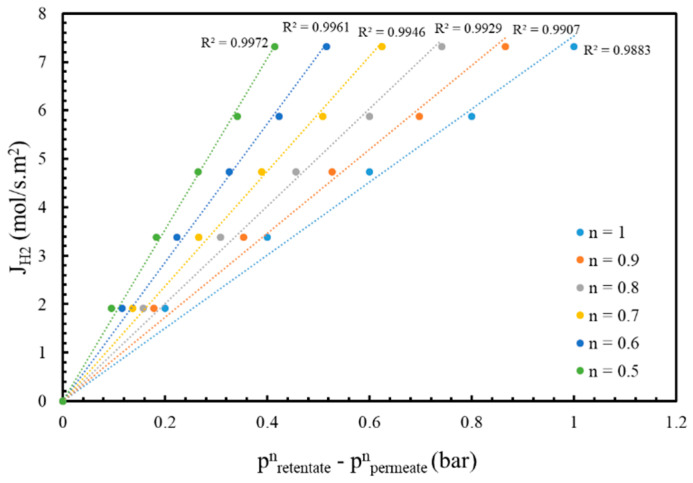
Hydrogen permeation flux vs different pressures and ‘n’ value at 500 °C.

This section evaluates the permeation properties of the Pd/YSZ membrane, including the H_2_ permeation flux, H_2_ permeance, and the ‘n’ value. According to Equation (9), the ‘n’ value was determined by plotting the hydrogen permeation flux against the driving forces for different ‘n’ values ranging from 0.5 to 1. A linear regression analysis was then used to determine the highest R^2^-value, which corresponds to the optimal ‘n’ value. Therefore, tests were performed at 500 °C, and the results were plotted in Figure 2, which indicated that the ‘n’ value was equal to 0.5. Permeation tests were also performed at higher temperatures of 550 °C and 600 °C, and the n value was confirmed to be 0.5. Moreover, the hydrogen permeation flux increased with temperature, consistent with the Arrhenius-type behavior described by Equation (10). As shown in Table 2, the hydrogen permeance of this membrane was compared to the values reported in the literature, and the results from this study are in good agreement with previously reported data. The apparent activation energy and pre-exponential factor were determined to be 23.4 kJ/mol and 5.7·10^−5^ mol·m^−2^·s^−1^·Pa^−1^, respectively, which are consistent with the literature, shown in Table 3.

### 3.2. MR Performance

**Figure 3:**
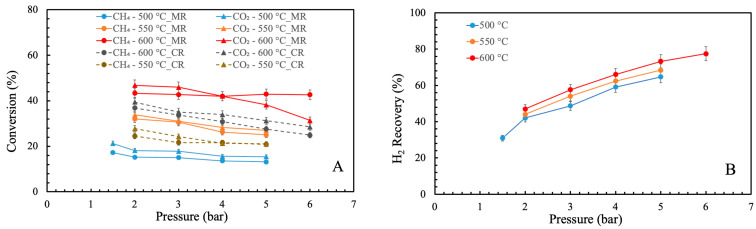
(**A**) CH_4_ and CO_2_ conversion and (**B**) H_2_ recovery vs. pressure and temperature.

The BDR tests were conducted with a Ru/CeO_2_ catalyst to evaluate the performance of the Pd/YSZ MR. The same tests were repeated by using a CR. Figure 3A,B show the CH_4_ and CO_2_ conversions, and hydrogen recovery at different temperatures and pressures. As seen in Figure 3A, the Pd/YSZ MR has shown better performance in terms of CH_4_ and CO_2_ conversion in comparison with CR, due to the removal of hydrogen from the reaction zone, which shifts the reaction towards the consumption of more reactants. The MR indicated higher CH_4_ conversion by 8% at 550 °C and 7% at 600 °C, and CO_2_ conversion by 6% at 550 °C and 7% at 600 °C than CR at 2 bar. Also, the CH_4_ and CO_2_ conversions improved with the increase in temperature. Elevated temperature positively influences the system through different mechanisms: (1) the BDR reaction is endothermic, (2) catalyst activity increases at higher temperatures, and (3) elevated temperature facilitates hydrogen permeation, shifting the reaction towards the consumption of more reactants [32,33]. At 2 bar, the CH_4_ and CO_2_ conversions increased from 15.2% and 18.1% at 500 °C to 43% and 46.7% at 600 °C. At low temperature and at each pressure, CO_2_ conversion was higher than CH_4_ conversion due to the catalyst’s selectivity towards CO_2_. A similar trend has been reported in the previous studies [32,33,39]. However, at 600 °C and elevated pressure of 5 and 6 bar, CH_4_ showed higher conversion than CO_2_ due to the methane decomposition. Under these conditions, increased coke formation was observed. The amount of deposited carbon increased from approximately 0.5 g at 550 °C to 1.2 g at 600 °C, confirming the enhanced coking tendency at higher temperatures. The pressure increase has a dual impact on the BDR reaction in the MR, contributing both positive and negative effects on system performance. The positive effect is due to the presence of the membrane: the higher the pressure, the higher the hydrogen removal from the reaction zone, which shifts the reaction towards the consumption of more reactants. In contrast, the negative effect is due to the intrinsic thermodynamics of the BDR reaction, as the overall reaction takes place with an increase in mole number, which makes the process less favorable at elevated pressures, thereby limiting the conversion. In Figure 3A it is visible that the increase in pressure caused a slight reduction in both CH_4_ and CO_2_ conversion, indicating that the negative effect of thermodynamics was more dominant than the positive effect of the membrane. For example, the CH_4_ and CO_2_ conversion decreased from 32% and 34% at 2 bar to 25% and 27% at 5 bar, at 550 °C. Notably, the CH_4_ conversion did not decline above 5 bar at 600 °C and remained constant due to the increased occurrence of methane decomposition reactions, which was also favored by the presence of the membrane.

Another important parameter used to evaluate the performance of the MR is the hydrogen recovery. Figure 3B shows the hydrogen recovery in the MR at different temperatures and pressures. As seen, the increase in temperature and pressure positively affect the hydrogen recovery through the MR due to Equations (9) and (10). In addition, by increasing the temperature, the conversion of the reactants was improved at each pressure, due to both membrane and thermodynamic effects. At 600 °C, the hydrogen recovery was enhanced from 47% at 2 bar to 78% at 6 bar. This trend is consistent with what is reported in the literature [41,42].

Table 4 summarizes reported performances of Pd- and Pd–Ag-based MRs for BDR using various catalysts and operating conditions. Overall, CH_4_ conversions in the literature range from 10% to 50%, with H_2_ recoveries up to 62%, depending on membrane thickness, temperature, and pressure. In this work, a 13 μm Pd membrane coupled with a Ru/CeO_2_ catalyst achieved CH_4_ and CO_2_ conversions of 43% and 32%, respectively, and H_2_ recovery of 78% at 600 °C and 6 bar.

**Figure 4:**
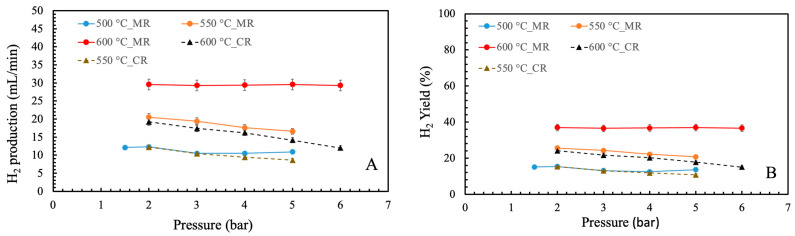
(**A**) H_2_ production, (**B**) H_2_ yield vs. pressure and temperature.

Figure 4A,B indicate the hydrogen production and yield against temperature and pressure. As can be seen, their trends are similar to CH_4_ conversion. The hydrogen production and yield were enhanced by an increase of temperature. For instance, at 2 bar the hydrogen production and yield were enhanced from 12.3 mL/min and 15.4% at 500 °C to 29.6 mL/min and 37% at 600 °C. However, they decreased with the increase of pressure due to a lower reactant conversion. In contrast, they both remained constant with the increase of pressure at 600 °C. The MR indicated higher hydrogen production and yield than CR when tests were performed at the same operating conditions. For example, hydrogen production and yield were 20.5 mL/min and 25.6% in MR, while they were 12.2 mL/min and 15.3%, respectively.

**Figure 5:**
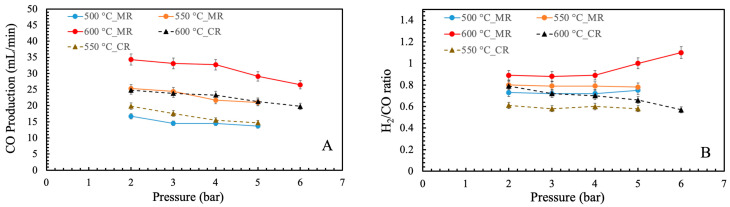
(**A**) CO production and (**B**) H_2_/CO ratio vs. pressure and temperature.

Figure 5A,B indicate CO production and the H_2_/CO ratio. The CO production was enhanced by the increase in temperature due to an improvement in the reactant conversion, while it declined with increasing pressure because of the reduction in conversion. Furthermore, CO production was lower in CR than in MR at similar operating conditions. This occurred due to lower CO_2_ conversion in the CR compared to the MR. Another key parameter in evaluating BDR performance is the H_2_/CO ratio, as it determines the suitability of the produced syngas for downstream applications. For instance, methanol synthesis requires an H_2_/CO ratio of approximately 2:1, whereas formaldehyde production typically operates near a 1:1 ratio. In this study, the H_2_/CO ratio increased with temperature, rising from 0.7 at 500 °C to 0.9 at 600 °C and 2 bar. At 600 °C and 6 bar, the ratio further approached unity, indicating that these operating conditions are favorable for generating syngas compositions suitable for formaldehyde synthesis. Moreover, MR exhibited a higher H_2_/CO ratio than the CR, reflecting the enhanced methane conversion and effective hydrogen removal achieved in the MR configuration. Finally, stable performance was achieved over multiple experiments conducted across a 30-day period.

### 3.3. Comparison of Ru/CeO_2_ and Ni/Al_2_O_3_ Catalyst

**Figure 6:**
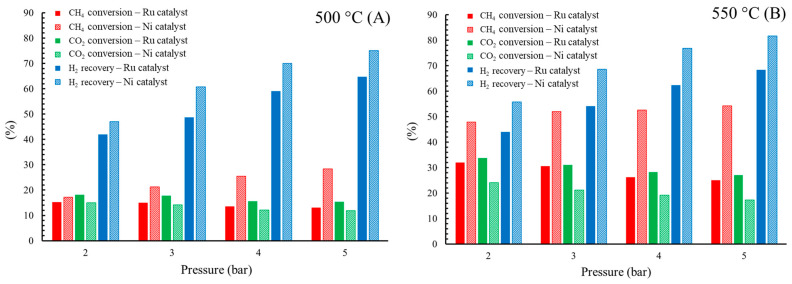
Comparison of Ru/CeO_2_ and Ni/Al_2_O_3_ catalyst in Pd/YSZ MR at (**A**) 500 °C and (**B**) 550 °C.

In this work, the Ru/CeO_2_ catalyst results were compared with those of a Ni/Al_2_O_3_ catalyst previously reported by the authors [15] in a Pd/YSZ MR under the same operating conditions. Figure 6 compares the catalytic performance of the Ru/CeO_2_ and Ni/Al_2_O_3_ systems in the Pd/YSZ MR. Overall, the Ni/Al_2_O_3_ catalyst exhibited higher CH_4_ conversion and hydrogen recovery, whereas the Ru/CeO_2_ catalyst achieved greater CO_2_ conversion at similar operating conditions. At 550 °C, the CH_4_ conversion obtained with the Ni/Al_2_O_3_ was approximately 20% higher than that with Ru/CeO_2_, reflecting the superior ability of Ni to activate C–H bonds and promote methane reforming pathways, thereby enhancing hydrogen generation and permeation through the membrane. In contrast, the Ru/CeO_2_ catalyst displayed lower CH_4_ conversion but significantly reduced carbon formation. This behavior can be attributed to the intrinsic properties of Ru combined with the oxygen storage and transfer capacity of CeO_2_, which collectively mitigate coke-forming reactions such as methane decomposition. The basicity of the CeO_2_ support further contributes to carbon suppression by favoring gasification of surface carbon species and maintaining active metallic sites. As a result, the Ru/CeO_2_ system demonstrated enhanced resistance to coking and improved long-term stability. The influence of pressure also differed between the two catalytic systems. For the Ni/Al_2_O_3_, CH_4_ conversion increased with pressure, indicating that methane decomposition was the dominant reaction pathway and that hydrogen removal through the membrane shifted the equilibrium toward further CH_4_ consumption. In contrast, the Ru/CeO_2_ catalyst exhibited a decrease in CH_4_ conversion with increasing pressure, consistent with the thermodynamic inhibition of the BDR reaction at elevated pressures. Nevertheless, the Ru/CeO_2_ catalyst maintained stable performance and minimized carbon deposition, confirming its suitability for sustained operation in membrane-assisted biogas reforming processes.

In terms of hydrogen recovery, the MR with the Ni catalyst indicated higher hydrogen recovery than the Ru catalyst due to greater methane conversion. On average, the hydrogen recovery was higher by 5% and 10% for 500 and 550, respectively. Additionally, the difference increases with higher pressure.

Furthermore, the results of Ru/CeO_2_ and Ni/Al_2_O_3_ catalysts are compared in Table 5 and Table 6 in terms of hydrogen production, hydrogen yield, CO production, and H_2_/CO ratio at similar operating conditions. The Ni/Al_2_O_3_ catalyst showed higher hydrogen production and yield due to greater methane conversion, with the difference between the two systems increasing at higher temperatures and pressures as methane decomposition became more dominant. In contrast, the Ru/CeO_2_ catalyst produced more CO, consistent with its tendency to promote the dry reforming pathway while suppressing coke-forming reactions. Finally, the amount of coke formation was significantly reduced by using Ru/CeO_2_ rather than Ni/Al_2_O_3_. For example, around 3 g of coke was produced by Ni/Al_2_O_3_ catalyst at 550 °C, but the coke formation was 0.5 g for Ru/CeO_2_ at similar conditions under 3 h for each reaction test.

From a catalysis point of view for dry reforming reaction, the elementary reaction steps differ significantly between Ni and Ru catalysts, leading to different catalytic performance. On Ni surfaces, methane activation proceeds through sequential C–H bond cleavage, with the first C–H dissociation identified as the rate-limiting step and associated with relatively high energy barriers. While Ni effectively dissociates CH_4_, it binds carbonaceous intermediates strongly, favoring their complete dissociation to surface carbon (C*). In parallel, CO_2_ activation on Ni is less efficient, occurring primarily at defect sites with higher energy barriers, which limits the supply of O* species required for carbon oxidation (C* + O* = CO*). This difference between rapid methane decomposition and sluggish CO_2_ dissociation results in significant carbon accumulation and catalyst deactivation. In contrast, Ru catalysts exhibit lower barriers for CH_4_ activation, facilitating faster C–H bond and easier hydrogen evolution, while also enabling more favorable CO_2_ activation through stronger Ru–O interactions that promote CO_2_* intermediates and enhance O* formation. The greater availability of reactive oxygen on Ru surfaces enhances carbon oxidation, thereby suppressing coke formation and maintaining long-term stability [55,56,57,58].

Moreover, Al_2_O_3_ and CeO_2_ supports have different performances in dry reforming reaction due to their different physicochemical properties. Al_2_O_3_ is largely inert toward CO_2_ activation and exhibits poor oxygen mobility. As a result, Ni/Al_2_O_3_ catalysts often suffer from rapid carbon accumulation and strong metal–support interactions, which can lead to reduced active site availability. In contrast, CeO_2_ provides unique redox properties through the reversible Ce^4+^/Ce^3+^ cycle, enabling oxygen vacancy formation and high oxygen storage capacity. These features facilitate CO_2_ activation and in-situ carbon oxidation (C* + O* = CO*), thereby enhancing coke resistance and catalyst stability [59,60,61,62,63].

## 4. Conclusions

In this study, the performance of a Pd/YSZ MR integrated with a Ru/CeO_2_ catalyst was evaluated under various operating conditions. The Pd/YSZ MR exhibited significantly higher methane conversion, up to 10% greater, compared to a CR under similar conditions. The results demonstrated that CH_4_ and CO_2_ conversions increased with temperature but decreased with pressure. The highest conversions of CH_4_ and CO_2_ were achieved, 43% and 46.7% at 2 bar, 600 °C, and a GHSV of 800 h^−1^. A maximum hydrogen recovery of 78% was observed at 6 bar, 600 °C, and 800 h^−1^. Hydrogen production and yield followed trends consistent with reactant conversions.

A comparative study between Ru/CeO_2_ and Ni/Al_2_O_3_ catalysts revealed that the Ni-based catalyst provided higher methane conversion but also led to coke formation. In contrast, the Ru/CeO_2_ catalyst effectively suppressed coke formation under identical conditions, indicating that the combination of Ru and CeO_2_ inhibits pathways responsible for carbon deposition.

## Figures and Tables

**Table 1:** Operating conditions for BDR reaction tests.

Operating Conditions
CH_4_/CO_2_ molar ratio = 1/1
Pressure (bar) = 1–6
Temperature (°C) = 500–600
GHSV* (h^−1^) = 800
Catalyst mass (g) = 5.4
Total feed flow rate (mL/min) = 80
Sweep gas flow rate (mL/min) = 30
Sweep gas = N_2_

**Table 2:** H_2_ permeance results comparison with the literature.

Membrane	Thickness (μm)	Preparation Method	T (°C)	H_2_ Permeance (mol.m^−2^.s^−1^.Pa^−1^)	Ref
Pd/TiO_2_	13	ELP	375	1.6 × 10^−6^	[44]
Pd/PSS	6	ELP	550	2 × 10^−6^	[45]
Pd/SiO_2_/PSS	6	ELP	500	2.7 × 10^−6^	[46]
Pd/PSS	19	ELP	450	1.1 × 10^−6^	[47]
Pd/YSZ	13	ELP	500	1.4 × 10^−6^	This work
Pd/YSZ	13	ELP	550	1.7 × 10^−6^	This work
Pd/YSZ	13	ELP	600	1.8 × 10^−6^	This work

**Table 3:** Ea comparison with the literature.

Membrane	Thickness (μm)	Ea (kJ/mol)	Ref
Pd/Al_2_O_3_	4.5	18.3	[46]
Pd/PSS	20	16.4	[48]
Pd/PSS	10	14.7	[49]
Pd/TiO_2_	0.4	21.2	[50]
Pd/Al_2_O_3_	6	18.5	[51]
Pd/YSZ	13	23.4	This work

**Table 4:** Results of this work compared with the literature.

Membrane	Thickness (μm)	Catalyst	T (°C)	P (Bar)	CH_4_ Conversion (%)	CO_2_ Conversion (%)	H_2_ Recovery (%)	Ref
Pd	22	Ru/ZrO_2_/La_2_O_3_	400	1	10	-	-	[33]
Pd	22	Ru/ZrO_2_/La_2_O_3_	450	1	26	-	-	[33]
Pd–Ag	100	Ru/Al_2_O_3_	500	2	22	-	17	[42]
Pd	20	Rh/La_2_O_3_	450	1	17	-	47	[41]
Pd	20	Rh/La_2_O_3_	500	1	25	-	60	[41]
Pd–Ag	23	Rh/La_2_O_3_	450	1	15	-	62	[41]
Pd–Ag	5	Ru/Al_2_O_3_	650	8	50	20	-	[52]
Pd–Ag	50	Pt/CeZrO_2_/Al_2_O_3_	550	1	44	-	-	[53]
Pd–Ag	50	Rh/La_2_O_3_	550	1	40	-	-	[54]
Pd	13	Ru/CeO_2_	500	2	15	18	42	This work
Pd	13	Ru/CeO_2_	600	6	43	32	78	This work

The - symbol is used to indicate that CH_4_ and CO_2_ conversion values are not reported in the referenced work.

**Table 5:** Comparison of Ru/CeO_2_ and Ni/Al_2_O_3_ catalyst in Pd/YSZ MR at 500 °C.

Pressure	H_2_ Production (mL/min)		CO Production (mL/min)		H_2_ Yield (%)		H_2_/CO Ratio	
	Ni	Ru	Ni	Ru	Ni	Ru	Ni	Ru
**2**	14.4	12.3	12	16.7	19	15.4	1.2	0.73
**3**	15.1	10.5	10.1	14.5	19.9	13.1	1.5	0.72
**4**	16.6	10.5	8.9	14.4	21.8	12.5	1.9	0.72
**5**	17	10.9	8.6	13.7	22.3	13.2	2	0.75

**Table 6:** Comparison of Ru/CeO_2_ and Ni/Al_2_O_3_ catalyst in Pd/YSZ MR at 550 °C.

Pressure	H_2_ Production (mL/min)		CO Production (mL/min)		H_2_ Yield (%)		H_2_/CO Ratio	
	Ni	Ru	Ni	Ru	Ni	Ru	Ni	Ru
**2**	30.1	20.5	12	25.3	39.6	25.6	2.5	0.8
**3**	33	19.4	11	24.4	43.3	24.2	3	0.79
**4**	33.9	17.6	9.9	21.7	44.6	22.1	3.4	0.78
**5**	35.1	16.6	9	21	46.2	20.7	3.9	0.78

## Data Availability

The original contributions presented in the study are included in the article. Further inquiries can be directed to the corresponding author.

## Abbreviations

BDR	biogas dry reforming
CR	conventional reactor
ELP	electroless plating
GHGs	greenhouse gases
GHSV	gas hourly space velocity
MR	membrane reactor
MDR	methane dry reforming
R	universal gas constant
SMR	steam methane reforming
**Symbols**	
E_a_	apparent activation energy
J_H_2__	hydrogen permeating flux
n	dependence factor
Pe_H_2__	hydrogen permeability
$p_{H_{2},\mathrm{permeate}}^{n}$	partial pressures in the permeate side
$p_{H_{2},\mathrm{retentate}}^{n}$	partial pressure in the retentate side
P_H_2__	hydrogen permeance
Pe^0^	pre-exponential factor
δ	membrane thickness
α	ideal selectivity
